# Toxicities of Chimeric Antigen Receptor T Cell Therapy in Multiple Myeloma: An Overview of Experience From Clinical Trials, Pathophysiology, and Management Strategies

**DOI:** 10.3389/fimmu.2020.620312

**Published:** 2020-12-23

**Authors:** Xiang Zhou, Leo Rasche, K. Martin Kortüm, Sophia Danhof, Michael Hudecek, Hermann Einsele

**Affiliations:** Department of Internal Medicine II, University Hospital of Würzburg, Würzburg, Germany

**Keywords:** CAR T cell, clinical trial, multiple myeloma, toxicity, pathophysiology, management

## Abstract

In the last few years, monoclonal antibodies (mAbs) such as elotuzumab and daratutumab have brought the treatment of multiple myeloma (MM) into the new era of immunotherapy. More recently, chimeric antigen receptor (CAR) modified T cell, a novel cellular immunotherapy, has been developed for treatment of relapsed/refractory (RR) MM, and early phase clinical trials have shown promising efficacy of CAR T cell therapy. Many patients with end stage RRMM regard CAR T cell therapy as their “last chance” and a “hope of cure”. However, severe adverse events (AEs) and even toxic death related to CAR T cell therapy have been observed. The management of AEs related to CAR T cell therapy represents a new challenge, as the pathophysiology is not fully understood and there is still no well-established standard of management. With regard to CAR T cell associated toxicities in MM, in this review, we will provide an overview of experience from clinical trials, pathophysiology, and management strategies.

## Introduction

Multiple myeloma (MM), a plasma cell neoplasm, is characterized by uncontrolled proliferation of clonal, malignant plasma cells in the bone marrow (1). Worldwide, MM accounts for approximately 10% of all hematological malignancies, and represents the second most common malignant hematological disease in adults with the majority of the patients being male and elder than 60 years of age (2–4). The survival outcome of patients with MM has been improved dramatically in the last few decades with the introduction of new treatments such as proteasome inhibitors (PIs), immunomodulatory drugs (IMiDs), and high-dose melphalan with autologous stem cell transplant (SCT) (5, 6). However, MM is still an incurable malignant disease as the majority of the patients with MM relapse in the course of the disease (7).

In the last few years, monoclonal antibodies (mAbs) such as elotuzumab and daratumumab have brought the management of MM into the new era of immunotherapy. So far, mAb containing therapy regimens have become the standard of care in patients with relapsed/refractory (RR) or newly diagnosed (ND) MM (8). More recently, immunotherapeutic strategies utilizing patients’ endogeneous T cells, such as bispecific antibodies (bsAbs) and chimeric antigen receptor (CAR) modified T cell therapy have shown promising efficacy in patients with RRMM in diverse clinical trials (9, 10). Preliminary results of some B cell maturation antigen (BCMA) targeted CAR T cell therapy trials have even demonstrated an overall response rate (ORR) of up to 100% in RRMM patients (11–13). For this reason, many patients with end stage RRMM regard CAR T cell therapy as their “last chance” and a “hope of cure”. Consequently, competitive enrollment and limited number of available slots represent major limitations of current CAR T cell trials for RRMM at many centers. In brief, CAR T cell therapy is highly effective and seems to be an attractive therapy option for MM patients. However, severe treatment–related adverse events (AEs) and even toxic death have also been observed in patients who have received CAR T cell therapy (14). The CAR T cell–related toxicity has posed a great challenge, as the mechanism is not fully understood and there is still no well-established standard of management strategy.

With respect to toxicities related to CAR T cell therapy in MM patients, this review will provide an overview of experience from clinical trials, pathophysiology, and management strategies.

## Overview of CAR T Cell Therapy for Multiple Myeloma

Mechanism of action, CAR targets, preclinical and clinical data on CAR T cell therapy for MM have been extensively discussed in previous review articles (15–17), and these issues are not the main topic of our current review. Here, we will just provide a brief overview.

T cells is an important element in adaptive immune system against tumor cells and external pathogens. The concept of CAR T cell therapy is to facilitate an interaction between tumor cell and patient’s own T cell. Using viral vector or electroporation, the CAR gene can be transmitted and integrated into the genome of autologous T cells, resulting in CAR expression on the cell surface (18). A CAR consists of an extracellular domain that can recognize tumor specific surface antigens and intracellular signaling (i.e., CD3ζ) or costimulatory domains (e.g., CD28 and/or 4-1BB), which promote T cell activation and proliferation (19, 20). In August 2017, the United States Food and Drug Administration (FDA) has approved the first CAR T cell therapy “Tisagenlecleucel”, a CD19 specific CAR construct, for the treatment of patients with RR B cell precursor acute lymphoblastic leukemia (ALL) (21). At present, diverse CAR T cell products for MM patients are under investigation within clinical trials.

**Figure 1:**
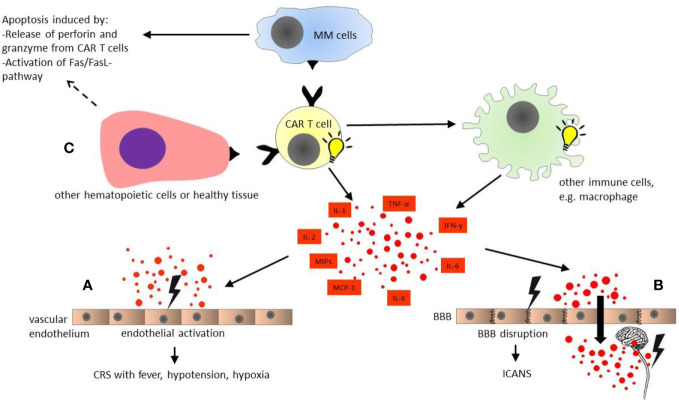
Pathophysiology of chimeric antigen receptor modified (CAR) T cell toxicities. CAR T cells are activated upon antigen recognition, and induce apoptosis of multiple myeloma cells by activation of Fas/FasL-pathway and releasing cytotoxic granules containing perforin and granzyme. In turn, CAR T cells activate other immune cells such as macrophages, which produce multiple cytokines simultaneously with activated CAR T cells themselves. **(A)** Cytokine release syndrome (CRS): The diverse cytokines cause activation of vascular endothelium. The endothelial activation plays a major role in cytokine release syndrome with fever, hypotension, and hypoxia. **(B)** Immune effector cell associated neurotoxicity syndrome (ICANS): The endothelial activation by multiple cytokines in blood stream results in disruption of blood-brain barrier. Subsequently, the central nervous system (CNS) is directly exposed to the cytokines in high concentrations, leading to local inflammation and secondary cytokine production by CNS itself, e.g., microglia. **(C)** On-target off-tumor toxicity: Healthy tissue and some other hematopoietic cells such as B cells also express the target antigen of CAR T cells. Hence, on-target off-tumor toxicities might occur, and are dependent on the selected CAR T cell target. All organ systems could be affected. BBB, blood-brain barrier; CAR T cell, chimeric antigen receptor modified T cell; CRS, cytokine release syndrome; ICANS, immune effector cell associated neurotoxicity syndrome; IL, interleukin; IFN, interferon; MCP, monocyte chemoattractant protein; MIPs, macrophage inflammatory proteins; MM, multiple myeloma; TNF, tumor necrosis factor.

CAR T cell is not an off-the-shelf product. Patients’ autologous T cells must be collected by leukapheresis and genetically modified to express CARs. As the currently available CAR T cell therapy trials include only patients with RRMM, a bridging therapy is usually needed to avert fulminant disease progression during the period between leukapheresis and CAR T cell infusion (22). The patients then receive lymphodepleting conditioning (LDC) to build up a favorable environment for CAR T cell activation, proliferation and survival, by multiple mechanisms including elimination of immunosuppressive cells and homeostatic cytokine sinks (23–25). Thirty to sixty minutes before CAR T cell infusion, pre-medication with acetaminophen and diphenhydramine should be given (26). CAR T cells bind to the target antigen shortly after the infusion, which leads to rapid *in vivo* activation and proliferation of CAR T cells (27). These cells show their cytotoxic activity by releasing cytotoxic granules containing perforin and granzyme, activation of the Fas and Fas ligand pathway, and production of multiple cytokines (28) (**Figure 1**).

Currently, BCMA represents the most commonly used CAR target in clinical trials investigating CAR T cell therapy for MM. BCMA, a transmembrane glycoprotein also referred to as CD269 or tumor necrosis factor receptor superfamily 17 (TNFRSF17), is highly expressed by malignant plasma cells (29, 30). More importantly, BCMA is almost absent in other cell lineages and normal human tissues (9). The expression of BCMA can promote myeloma growth and protect MM cells from apoptosis (31–33). A recent updated meta-analysis of 20 studies demonstrated a pooled ORR of 84% with 43% complete remission (CR) in patients with heavily pretreated RRMM who had received BCMA directed CAR T cell (10). Importantly, even the heavily pretreated patients with extramedullary disease (EMD), a high risk feature, presented a high ORR of 78%, which could not be achieved by conventional combination chemotherapies such as “VDT-PACE” (bortezomib, dexamethasone, thalidomide, cisplatin, doxorubicin, cyclophosphamide, and etoposide) (34), “DexaBEAM” (dexamethasone, carmustine, etoposide, cytarabine, and melphalan) (35), daratumumab (36) or carfilzomib containing treatments (37). However, as reported by Gagelmann et al., synthesized results of five full publications from China or the United States (38–42) yielded a relapse rate of 45% at the last follow up, and the median progression-free survival (PFS) was only 10 months (10).

In principle, other antigens, which are presented by malignant plasma cells, can likewise be selected as CAR T cell target for MM patients. CAR T constructs targeting alternative antigens such as CD138 (syndecan-1) (43), CD19 (44), CD38 (45), kappa light chain (46), signaling lymphocyte activation molecule family 7 (SLAMF7, CS1, or CD319) (47), G protein coupled receptor family C group 5 member D (GPRC5D) (48), CD44v6 (49), and natural killer group 2D (NKG2D) (50) also have been explored in preclinical settings and are presently under clinical investigation. Besides these, some other clinical trials evaluating multi-specific CAR T cell therapy targeting BCMA and an additional antigen, e.g., CD38 (51), SLAMF7 (52), transmembrane activator and calcium modulator and cyclophilin ligand interactor (TACI) (53), and CD19 (54), are ongoing. Preliminary results from the phase 1 trial at the Wuhan Union Hospital, China, demonstrated a high ORR of 87.5% (14/16) in heavily pretreated RRMM patients who received BCMA/CD38 bispecific CAR T cells, with all five patients with EMD responding to this therapy (54). We summarize the currently available clinical data on CAR T cell therapy in RRMM in **Table 1**.

**Table 1:** Selected clinical trials of CAR T cell therapy in relapsed/refractory multiple myeloma (published as manuscript or abstract).

Target	Identifier	Product	Phase	N	LDC	Dose (cells/kg)	Prior lines of therapy	ORR	Median PFS	Toxicity	Management of CAR T cell therapy associated toxicities	Ref
BCMA	NCT02215967	CAR-BCMA	1	24	Cy/Flu	0.3–9 × 10^6^	9.5	81%^#^	31 weeks	CRS (≥G3) = 6 pts, CRS (G1–2) = 7 pts, cytopenia (≥G3) = 15 pts, severe NT = 1 pt	tocilizumab in 5 pts, corticosteroids in 4 pts	(38)
			1	12	Cy/Flu	0.3–9 × 10^6^	7	100%^#^	NR	cytopenia (≥G3) = 12 pts, CRS (all Gs) = 6 pts	tocilizumab in 2 pts	(55)
	NCT02546167	CART-BCMA	1	25	Cy or none	cohorts 1 and 3: 1–5 × 10^8^, cohort 2: 1–5 × 10^7^	7	cohort 1: 44%, cohort 2: 20%, cohort 3: 64%	cohort 1: 65 days, cohort 2: 57 days, cohort 3: 125 days	CRS (all Gs) = 88%, NT = 32%	tocilizumab in 6 pts, siltuximab in 1 pt	(41)
	NCT02658929	bb2121	1	33	Cy/Flu	50×, 150×, 450×, or 800×10^6^	7–8	85%	11.8 months	neutropenia (≥G3) = 85%, CRS (all Gs) = 76%, CRS (≥G3) = 6%, NT (all Gs) = 42%, NT (G4) = 3%	tocilizumab in 7 pts, corticosteroids in 4 pts	(39)
	NCT03090659	LCAR-B38M	1	57	Cy	0.07–2.1 × 10^6^	3	88%	15 months	leukopenia (≥G3) = 30%, CRS (all Gs) = 90%, CRS (≥G3) = 7%, NT (G1) = 2%	tocilizumab in 26 pts	(42)
			1	17	Cy/Flu or Cy	0.21–1.52 × 10^6^	4	88%	12 months	CRS (G1–2) = 59%, CRS (≥G3) = 41%(1G5) cytopenias (all Gs) = 82%	tocilizumab in 10 pts (with additional etanercept in 2 pts)	(40)
	NCT03430011	JCARH125	1/2	19	Cy/Flu	50 × 10^6^ or 150 × 10^6^	10	100%	NR	CRS (G1–2) = 6 pts, N T(all Gs) = 3 pts, sepsis after LDC (G5) = 1 pt	tocilizumab in 1 pt, corticosteroids in 1 pt	(56)
			1/2	51	Cy/Flu	300×, 450×, or 600×10^6^	6	91%	NR	CRS (G ≥ 3) = 2%, NT (G ≥ 3) = 4%, infection (G ≥ 3) = 14%,	tocilizumab and/or corticosteroids in 40 pts, anakinra in 7 pts	(57)
	NCT03070327	MCARH171	1	11	Cy/Flu or Cy	72×, 137×, 475×, or 818 × 10^6^	6	64%	NR	CRS (G1–2) = 40%, CRS (G3) = 20%, NT (G2) = 10%	tocilizumab in 3 pts	(58)
	NCT03338972	FCARH143	1	7	Cy/Flu	5× or 15 × 10^7^	8	100%	NR	CRS (G1–2) = 86%	NR	(13)
	NCT03288493	P-BCMA-101	1/2	25	Cy/Flu	0.5–5 × 10^8^	7	48%	NR	CRS (G ≥ 3) = 32%, NT (G ≥ 3) = 12%	NR	(59)
	NCT03274219	bb21217	1	22	Cy/Flu	150, 450, 800, or 1200 × 10^6^	7	83%	NR	CRS = 59% (5G1, 7G2,1G3), NT = 23% (1G1, 2G2,1G3,1G4)	tocilizumab and/or corticosteroids	(60)
	NCT03548207	JNJ-68284528	1b/2	29	Cy/Flu	median 0.73 × 10^6^	5	100%	NR	neutropenia (G ≥ 3) = 100%, CRS = 93% (25G1–2,1G3,1G5), NT = 14% (3G1–2,1G3)	NR	(61)
	NCT03361748	bb2121	2	128	Cy/Flu	150–450 × 10^6^	6	73%	8.6 months	cytopenias(all Gs) = 97%, CRS(all Gs) = 84%(5G3,1G4,1G5), NT(G3) = 18%	NR	(62)
	NCT03661554	NR	1	16	Cy/Flu	2–10 × 10^6^	NR	100%	NR	CRS (G3–4) = 2 pts, CRS (G0–2) = 14 pts	NR	(63)
	NCT03093168	NR	1	46	Cy/Flu	9 × 10^6^	NR	79.6%	15 months	CRS (G1–2) = 22.7%, CRS (G3) = 6.8%	NR	(64)
	NCT03716856 NCT03302403 NCT03380039	CT053	1	24	Cy/Flu	1.5×, 0.5×, 1×, or 1.8 × 10^8^	4.5	87.5%	NR	leukopenia (G ≥ 3) = 87.5%, CRS(G1–2) = 62.5% (3G1,12G2), NT = 12.5% (2G1,1G3), neutropenic infection (G5) = 1 pt	tocilizumab in 8 pts	(65)
CD138	NCT01886976	CART-138	1/2	5	PCD/CP/VAD	median 0.756 × 10^7^	10	0%	NR	fever (G3) = 80%	NR	(66)
CD19	NCT02135406	CTL019	1	10	Mel + ASCT	1–5 × 10^7^	6	90%	200 days	CRS (G1) = 1 pt, GvHD (G3) = 1 pt, mucositis (G3) = 1 pt	NR	(67)
NKG2D	NCT02203825	CM-CS1	1	5	None	1 × 10^6^–3 × 10^7^	≥5	0%	NR	no CRS, no NT, no treatment related toxicity (G ≥ 3)	NR	(50)
kappa light chain	NCT00881920	κ.CART	1	7	Cy or none	0.92–1.9 × 10^8^/m^2^	4	0%	NR	no CRS	NR	(46)
BCMA and CD19*	NCT03196414	NR	1/2	28	Cy/Flu	BCMA: 2–6.8 × 10^7^, CD19: 1 × 10^7^	3	92.6%	8 months	CRS (G1–2) = 19 pts, CRS (G3) = 7 pts, CRS (G4) = 2 pts, NT (G4) = 1 pt,	NR	(68)
BCMA and CD19*	NCT03455972	NR	1/2	32	BuCy or Mel + ASCT	CD19: 1 × 10^7^, BCMA: NR	NR	100%	NR	CRS (G1–2) = 31 pts, CRS (G3) = 1 pt	tocilizumab in 1 pt	(69)
BCMA and CD19*	ChiCTR-OIC-17011272	NR	2	22	Cy/Flu	CD19: 1 × 10^6^, BCMA: 1 × 10^6^	6	95%	VGPR: 243 days, sCR: 268 days	CRS (G1–2) = 18 pts, CRS (G ≥ 3) = 1 pt, cytopenias (all Gs) = 20 pts, NT (all Gs) = 2 pts, cerebral hemorrhage (G5) = 1 pt	NR	(70)
BCMA/CD38^ǂ^	ChiCTR180001814	BM38	1	16	Cy/Flu	0.5×, 1.0×, 2.0×, 3.0× or 4.0× 10^6^	NR	87.5%	NR	CRS (G1–2) = 10 pts, CRS G ≥ 3) = 4 pts	tocilizumab in 4 pts	(51)
BCMA/CD19^ǂ^	NR	NR	NR	5	Cy/Flu	1.0×10^6^ or 2.0×10^6^	3	100%	NR	CRS (G1) = 3 pts	NR	(54)
BCMA/TACI^ǂ^	NCT03287804	AUTO2	1/2	12	Cy/Flu	15×, 75×, 225×, 600× or 900× 10^6^	5	43%	NR	anemia (G ≥ 3) = 82%, neutropenia (G ≥ 3) = 73%, CRS (all G1) = 45%	tocilizumab in 3 pts	(53)

ASCT, autologous stem cell transplant; BCMA, B cell maturation antigen; Bu, busulfan; CAR T cell, chimeric antigen receptor modified T cell; CP, chlorambucil, prednisone; CRS, cytokine release syndrome; Cy, cyclophosphamide; Flu, fludarabine; G, grade; LDC, lymphodepleting conditioning; Mel, melphalan; NR, not reported; NT, neurotoxicity; ORR, overall response rate; PCD, pomalidomide, cyclophosphamide, dexamethasone; PFS, progression-free survival; pt, patient; Ref, reference; sCR, stringent complete remission; TACI, transmembrane activator and calcium modulator and cyclophilin ligand interactor; VAD, vincristine, doxorubicin, dexamethasone; VGPR, very good partial remission.

^#^In patients with the highest dose.

*The patients received both BCMA and CD19 directed CAR T cells.

^ǂ^Bispecific CAR T cells.

In brief, the currently available data suggest, even in patients with high-risk features, a superior efficacy of CAR T cell therapy in RRMM compared with already approved highly potent novel agents including carfilzomib, pomalidomide, and daratumumab. These results encourage further development and investigation of CAR T cell therapy in MM. CAR T cell therapy has the potential to become a new backbone of MM management and to be incorporated into the standard frontline treatment.

## CAR T Cell–Related Toxicity in Multiple Myeloma: Pathophysiology and Clinical Presentation

CAR T cell therapy is often associated with a prolonged cytopenia phase and excessive cytokine production (71, 72). In general, the severity of CAR T cell therapy associated toxicity is related to tumor burden, dose of CAR T cells, as well as the antigen that has been targeted. In MM patients, with BCMA being the most commonly used target antigen, clinical data on CAR T cell–related toxicity are mainly based on these studies. The most common toxicities include cytokine release syndrome (CRS), immune effector cell associated neurotoxicity syndrome (ICANS), and cytopenia-related complications, which have also been reported in studies investigating anti-CD19 CAR T cell therapy in B cell leukemia and/or non-Hodgkin’s lymphoma (NHL) (21, 72–76). However, severe CRS and/or ICANS are less common in MM than that in ALL or NHL, probably due to reduced T cell fitness in these heavily pretreated patients with RRMM. In addition, awareness of other on-target off-tumor side effects is also important in the clinical practice.

### Cytokine Release Syndrome

CRS is characterized by hyper-inflammatory immune response following CAR T cell infusion. The pathophysiology of CRS is not yet fully understood. Some potential mechanisms of CRS have been illustrated in **Figure 1**. After CAR T cell infusion, the immune interaction between CAR T and MM cells leads to CAR T cell activation and expansion, which subsequently causes massive cytokine production from CAR T cells, e.g., interferon-γ (IFN-γ), tumor necrosis factor α (TNF-α), and granulocyte/macrophage colony stimulating factor (GM-CSF) (77, 78). These T cell effector cytokines, in turn, result in activation of other immune or non-immune cells, e.g., the monocyte/macrophage system (79). In CRS, the macrophage is considered as the main source of the pro-inflammatory cytokines and/or mediators such as interleukin-1 (IL-1), interleukin-6 (IL-6), interleukin-10 (IL-10), IFN-γ, macrophage inflammatory proteins (MIP), monocyte chemoattractant protein-1 (MCP-1), as well as inducible nitric oxide synthase (iNOS), etc (80). Moreover, macrophage can also secrete catecholamines, which can in turn enhance the hyper-inflammatory immune response (81–83). Furthermore, experience with anti-CD19 CAR T cell therapy in B cell ALL has suggested that the cytokine storm can also result in endothelial activation, which is characterized by elevation of angiopoietin-2 (Ang-2) and von Willebrand factor (vWF) released from Weibel-Palade bodies of endothelium upon activation (71, 84). However, it is not always possible to measure the levels of all the involved cytokines in the clinical practice. In the routine laboratory examination, some serum biomarkers, e.g., C reactive protein (CRP) and ferritin are usually elevated in patients suffering from CRS (85). Although these laboratory markers are often unspecific, they could be used as surrogate markers to monitor the development of CRS and to evaluate the response to pharmacologic intervention (18).

Similar to ALL and/or NHL patients, CRS is also the most common AE in MM patients treated with CAR T cell therapy. As mentioned above, CRS incidence and severity are related with CAR T target. In MM, the patients receiving BCMA directed CAR T cells have shown a very high CRS rate of >80% (39–42), and toxic death due to severe CRS has also been observed in some BCMA CAR T cell trials (40, 61, 62). Similarly, in a CD138 targeted CAR T cell therapy study, Guo et al. reported that 80% (4/5) of the patients developed fever >39°C, which could also be interpreted as CRS (66). By contrast, CD19, NKG2D or kappa light chain targeted CAR T cells have shown a low CRS incidence or even no therapy-related toxicity at all, with these treatments being less effective than BCMA CAR T cells (46, 50, 67). In MM patients simultaneously receiving two different CAR T constructs, i.e., anti-BCMA and anti-CD19 CAR T cells, the CRS incidence is comparable to that of BCMA directed product cell alone (68–70). More recently, published data on BCMA/CD38 or BCMA/CD19 bispecific CAR T cell therapies have demonstrated a CRS rate similar to that in unispecific BCMA directed products, and the ORRs were >80% in these studies (51, 54). On the other hand, BCMA/TACI-targeted bispecific CAR T cells have yielded a low CRS rate of 45%, with the ORR being merely 43%. This trial is terminated, as preliminary efficacy has been determined as not sufficient to warrant further investigation (53). The currently available data of CRS in CAR T cell therapy in MM are shown in **Table 1**.

The onset time points and durations of CRS differ widely among the patients receiving different CAR T cell products. CRS usually occurs in the first week after the CAR T cell infusion, and can last a couple of days (86). Therefore, a close monitoring is mandatory during this period. As CRS is a systemic immune reaction, all organ systems could be affected. Typical early signs of CRS include fever ≥38°C, flu like symptoms, arthralgia, myalgia, and fatigue, which are mainly caused by INF-γ and TNF-α production by CAR T cells themselves (78, 87). Additionally, hypoxia, hypotension, and end organ damages such as liver function abnormalities, coagulopathy, decompensated heart failure, cardiac injury, and arrhythmia have already been reported in severe CRS, and CRS could develop into a life-threatening situation (42, 55). As previously mentioned, the excessive cytokine release from CAR T cells and/or other immune cells might cause endothelial activation, and might subsequently contribute to severe CRS with hemodynamic instability, capillary leak, and consumptive coagulopathy (71, 84). Since activated macrophages are considered as the main source of pro-inflammatory cytokines, secondary hemophagocytic lymphohistocytosis/macrophage activation syndrome (HLH/MAS) could be an accompanying event during CRS (88, 89). Indeed, some patients with CRS do meet the HLH-2004 diagnostic criteria (90).

In previous studies, CRS was assessed using different grading systems, e.g., Penn grading scale (91), Lee criteria (92), and CAR T cell therapy associated TOXicity Working Group (CARTOX) system (93) and, therefore, the incidence and severity of CRS cannot be directly compared among these studies (94). More recently, to solve this issue, the American Society for Transplantation and Cellular Therapy (ASTCT) developed a standardized CRS grading system, which was also recommended by the European Society for Blood and Marrow Transplantation (EBMT) (22, 95). In the ASTCT scale, CRS grading is based on presence of fever, hypoxia, and hypotension, with fever ≥38°C being present in all grades (95). We summarize the ASTCT grading system for CRS in **Table 2**.

**Table 2:** The American Society for Transplantation and Cellular Therapy (ASTCT) grading system and management strategy for cytokine release syndrome (CRS) [Table adapted from Yakoub-Agha et al. (22)].

	Grade 1	Grade 2	Grade 3	Grade 4
Fever ≥38°C	Yes	Yes	Yes	Yes
Hypotension	No	Yes, but does not require vasopressor	Yes, and requires vasopressor	Yes, and requires more than one vasopressors (excluding vasopressin)
Hypoxia	No	Yes, and requires only low-flow O_2_ ≤ 6 L/min (nasal cannula)	Yes, and requires high-flow O_2_ > 6 L/min (face mask, non-rebreather mask, venturi mask)	Yes, and requires positive pressure (CPAP, BiPAP, intubation, and mechanical ventilation)
ICU	Not required	Alert ICU	Transfer to ICU	Transfer to ICU
Investigations	Diagnostic tests for infections, e.g., blood cultures, laboratory examinations, and imaging			
Management	Symptomatic measures, anti-infective treatment as per institutional standards			
	Consider tocilizumab IV 8mg/kg, if the symptoms persist ≥3 days	Repeat tocilizumab or switch to siltuximab IV 11 mg/kg	Repeat tocilizumab or switch to siltuximab IV 11 mg/kg, and dexamethasone IV 10 mg every 6 h	Repeat tocilizumab or switch to siltuximab IV 11 mg/kg, and dexamethasone IV 20 mg every 6 h or methylprednisolone 1,000 mg/d. Consider other experimental salvage therapy options

BiPAP, Biphasic Positive Airway Pressure; CPAP, continuous positive airway pressure; ICU, intensive care unit; IV, intravenous.

### Immune Effector Cell-Associated Neurotoxicity Syndrome

ICANS, formerly CAR T cell–related encephalopathy syndrome (CRES), is another common AE related to CAR T cell therapy. As the name suggests, ICANS is a central nervous system (CNS) toxicity associated with immune cell activation. Presently, the mechanism of ICANS is not fully understood. There are some hypotheses based on data from anti-CD19 CAR T cell trials in B cell ALL (**Figure 1**). Previous studies have demonstrated a clear correlation of ICANS with the presence and severity of CRS (96, 97). As discussed above, upon CAR T cell activation, multiple cytokines such as IL-6, IFN-γ, and TNF-α released from CAR T cells and other immune cells might in turn induce endothelial activation (71, 84). Recent studies with anti-CD19 CAR T cells in ALL patients have suggested that blood-brain barrier (BBB) disruption following activation of vascular endothelium might play a major role in ICANS (96, 97). For instance, Santomasso et al. have reported that patients with ICANS have significantly increased cerebrospinal fluid (CSF) protein levels and CSF/serum albumin quotients after anti-CD19 CAR T cell treatment, probably due to BBB disruption, and CSF protein concentration correlated with the severity of ICANS. These findings support the hypothesis that BBB dysfunction might promote the development of ICANS (96). In this case, CSF and CNS are directly exposed to the excessive cytokine production in the blood stream (88). This is in line with the findings of Gust et al. that the concentrations of multiple cytokines such as IFN-γ, TNF-α, and IL-6 are comparable between serum and CSF in patients suffering from acute ICANS, with a cytokine gradient between CSF and serum being observed at baseline prior to LDC (97). Moreover, CNS cells like microglia can also be activated by the diverse cytokines migrated to CSF, triggering secondary CNS production of cytokines such as IL-6, interleukin-8 (IL-8), IFN-γ induced protein 10 (IP-10), and MCP-1, and consequently local inflammation (96). Furthermore, Santomasso et al. have also observed increased levels of endogenous excitatory N-methyl-D-aspartate (NMDA) receptor agonists quinolinic acid and glutamate in CSF during ICANS, probably contributing to excitotoxicity such as myoclonus or seizure (96). Altogether, ICANS is a multifactorial event, and further studies are needed to elucidate the underlying pathophysiology of ICANS.

Generally, ICANS is less common than CRS, and the vast majority of the patients with ICANS also present a CRS (98). Evidence of ICANS in MM patients is mainly based on clinical trials investigating BCMA targeted CAR T cells. Overall, data on ICANS incidence are very heterogeneous among the currently available clinical trials, with some studies reporting even no neurotoxicity (13, 26, 51). Ordinarily, ICANS appears simultaneously with CRS or shortly after its peak, while delayed ICANS onset after CRS resolution has also been observed (39, 71, 96, 97). ICANS is mostly reversible, and the duration of ICANS ranges from a couple of hours to a few weeks (39, 55, 56, 58, 62). Therefore, the patients need close monitoring for ICANS during the entire course of CAR T cell therapy. The treating physicians should be vigilant that late onset neurotoxicity might occur. On the other hand, MM patients treated with non-BCMA directed CAR T cells such as CD138, CD19, NKG2D, and kappa light chain have not shown any severe ICANS after the treatment, probably because these products can only achieve limited CAR T cell activation and clinical efficacy (46, 50, 66, 67). At present, experience with bispecific CAR T cell therapy in MM is still very limited, and the published data of BCMA/CD38, BCMA/TACI and BCMA/CD19 targeted CAR T cell studies have not demonstrated any treatment associated neurotoxicity (51, 53, 54). Further investigations of bispecific CAR T cell therapy in MM are needed at this point. We summarize the clinical data on ICANS in MM patients in **Table 1**.

The clinical presentation of ICANS is highly variable. In MM patients treated with anti-BCMA CAR T cells, typical signs of ICANS include confusion (38, 41), delirium (38, 55), transient aphasia (41, 42), encephalopathy (38, 41, 60, 70), bradyphrenia (39), agitation (42), hallucination (39), obtundation (41), seizure (41, 42, 68), mild cerebral edema in magnetic resonance imaging (MRI) (41), polyneuropathy/polymyopathy (38), tremor (39), dizziness (39, 60), and vertigo (60). Notably, the majority of patients have shown a mild neurotoxicity and, as of now, toxic death due to ICANS has not been reported in BCMA CAR T cell trials in patients with MM. In a study of Yan et al., one patient had received both anti-BCMA and anti-CD19 CAR T cells, and this patient died of thrombocytopenia-related cerebral hemorrhage, which was not classified as treatment-related neurotoxicity by the investigators (70). By contrast, extensive neurological defects and even toxic death due to cerebral edema were observed in anti-CD19 CAR T cell trials in leukemia and/or NHL (99–102). Importantly, it has been observed that ICANS is enriched in patients with a high tumor burden such as EMD and plasma cell leukemia (PCL) (39, 41, 56). Often, severe neurotoxicity is associated with elevated prothrombin time (PT), activated partial thromboplastin time (aPTT), D-dimer, and low fibrinogen (88). Taken together, ICANS in MM is mainly observed in patients treated with anti-BCMA CAR T cells, and has a similar symptom spectrum with less severity compared to that in anti-CD19 CAR T cell therapies for leukemia and/or NHL.

Previously, ICANS was graded according to Common Terminology Criteria for Adverse Events (CTCAE) criteria (103). In 2017, the CAR T cell therapy associated toxicity 10-point neurological assessment (CARTOX-10) score has been developed specifically for grading CAR T cell–related neurotoxicity (93). More recently, the ASTCT has published an ICANS grading system based on immune effector cell associated encephalopathy (ICE) score, depressed level of consciousness, presence of seizure, motor findings, and presence of elevated intracranial pressure (ICP) or cerebral edema, which represents the currently most commonly used tool for assessment of ICANS (95). We summarize the ASTCT criteria in **Table 3**. In brief, ICANS is primarily a clinical diagnosis, while neuroimaging and electroencephalography (EEG) should be performed to evaluate cerebral edema and seizure, respectively.

**Table 3:** The American Society for Transplantation and Cellular Therapy (ASTCT) grading system and management strategy for immune effector cell associated neurotoxicity syndrome (ICANS) for adults [Table adapted from Yakoub-Agha et al. (22) and Neill et al. (104)].

	Grade 1	Grade 2	Grade 3	Grade 4
ICE score*	7–9	3–6	0–2	Unable to perform
Depressed level of consciousness	Awakens spontaneously	Awakens to voice	Awakens only to tactile stimulus	Unarousable or requires vigorous or repetitive tactile stimuli to arouse. Stupor or coma
Seizure	No	No	Any clinical seizure that resolves rapidly or non-convulsive seizures on EEG that resolve with intervention	Life-threatening prolonged seizure (>5 min) or repetitive clinical or electrical seizures without return to baseline in between
Motor findings	No	No	No	Deep focal motor weakness such as hemiparesis or paraparesis
Elevated ICP/cerebral edema	No	No	Focal/local edema on neuroimaging	Diffuse cerebral edema on neuroimaging; decerebrate or decorticate posturing; or cranial nerve VI palsy; or papilledema; or Cushing’s triad^#^
ICU	Alert ICU	Transfer to ICU	Transfer to ICU	Transfer to ICU
Investigations	Neurological examinations including fundoscopy to exclude papilledema, EEG, MRI, and lumbar puncture in absence of contraindications			
Management	Alert neurologist, elevate the head of the patient’s bed to 30°, management of CRS if concurrent			
	Close monitoring	Dexamethasone IV 10 mg every 6 h, and consider levetiracetam 750 mg bid as prophylaxis for seizures	Dexamethasone IV 20 mg every 6 h. If seizure, clonazepam IV 1mg or other benzodiazepines to terminate it, then loading with levetiracetam	Management of seizure as per grade 3. If papilledema, start acetazolamide IV 1,000 mg followed by 250–1,000 mg bid. If elevated ICP/cerebral edema, consider hyperosmolar therapy with mannitol and hyperventilation. Methylprednisolone IV 1,000 mg/d. Evaluation of other experimental salvage options

CRS, cytokine release syndrome; EEG, electroencephalography; ICE, immune effector cell associated encephalopathy; ICP, intracranial pressure; ICU, intensive care unit; IV, intravenous; MRI, magnetic resonance imaging.

*Orientation to year, month, city, hospital: 4 points; Ability to name 3 objects: 3 points; Ability to follow simple commands: 1 point; Ability to write a standard sentence: 1 point; Ability to count backward from 100 by 10: 1 point.

^#^Irregular, decreased respirations, Bradycardia, Systolic hypertension.

Lately, Rubin et al. developed a model for predicting neurotoxicity after anti-CD19 CAR T cell therapy with axicabtagene ciloleucel for RR NHL. In this scoring system, the following factors were considered: age (≥52 versus <52 years), maximum CRP (≥8.95 versus <8.95 mg/dl), maximum ferritin (≥641 versus <641 ng/ml), minimum white blood cell (WBC) count (<790 versus ≥790/μl), time point of CRS onset (prior to versus after day 3), histologic subtype of lymphoma (aggressive versus indolent), temperature (≥38.5°C versus <38.5°C), presence of CRS of any grade, and use of tocilizumab prior to day 5 (105). This is a valuable instrument for triaging and resource allocation, and development of such a predictive model for MM patients is highly warranted.

### Cytopenia-Related Adverse Events and Other On-Target Off-Tumor Toxicities

In the most of the CAR T cell trials, patients receive LDC prior to CAR T cell infusion to create a favorable environment for CAR T cells (23–25). However, LDC is also associated with more frequent and more severe CRS and/or ICANS (89). Additionally, cytopenias, i.e., anemia, thrombocytopenia, leukopenia, and neutropenia, following LDC and/or CAR T cell infusion occur in the vast majority of the patients (**Table 1**). In MM patients who received anti-BCMA CAR T cells, toxic death due to neutropenic infection or cerebral hemorrhage was already reported (65, 70). There was also a patient who died of sepsis after LDC and, therefore, could not receive the CAR T cell infusion (56). Moreover, persisting cytopenia and even secondary myelodysplastic syndrome (MDS) have been observed in patients with RR ALL and/or NHL treated with anti-CD19 CAR T cells (106). At present, long-term follow up data in MM patients following CAR T cell therapy is still pending. MM patients who receive CAR T cell therapy are often heavily pretreated with tandem autologous SCT and/or multiple intensive immunochemotherapies, which can cause preexisting bone marrow toxicities as risk factor for sustained cytopenia. In summary, the treating physicians should be aware of acute cytopenia-related AEs such as infection and bleeding as well as delayed cytopenia and secondary hematological malignancies.

Another major issue of CAR T cell therapy is the so-called “on-target off-tumor” toxicity (**Figure 1**). As the CAR target might also present in other hematopoietic cells and healthy tissue, it is important to select a tumor-restricted antigen as CAR target (107). As previously discussed, BCMA is the most widely used target for cellular immunotherapy. It is highly expressed by mature B cells including plasma cells, and is almost absent in other cell lineages (32, 108, 109). However, the presence of BCMA on healthy plasma cells might lead to secondary hypogammaglobulinemia, since the healthy plasma cells can also be affected by CAR T cells. Similarly, anti-CD19 CAR T cell therapy can cause B cell aplasia through depletion of CD19 positive B cell progenitors (110). Moreover, CD38, another immune target for plasma cells, is also expressed in gastrointestinal tract, cerebellar Purkinje cells or even T cells themselves (111–113). Although the currently available BCMA/CD38 bispecific CAR T cell therapy has shown a similar safety profile as seen in BCMA directed products without any unexpected events (51), on-target off-tumor toxicity and fratricide cytotoxicity should be taken into account when targeting CD38 with CAR T cells. The same holds true for alternative CAR targets for MM, e.g., SLAMF7, CD138, and CD44v6, etc (114).. Furthermore, on-target off-tumor toxicity has also been considered as a potential mechanism of ICANS. Autopsy studies in patients, who were treated with anti-CD19 CAR T cells and died due to severe ICANS, revealed a significant CAR T cell infiltrate in the brain parenchyma and CSF, yielding the hypothesis that direct cell-cell interaction between CNS and CAR T cells might also have a role in the pathogenesis of ICANS (97). Indeed, on-target off-tumor toxicity poses a major concern in the development of CAR T cell therapy. The treating physicians should be aware of this potential toxicity in the clinical practice.

## Management of CAR T Cell–Related Toxicity

CAR T cell–related toxicity requires a multidisciplinary management, involving hematologist, neurologist, radiologist as well as intensive care unit (ICU). The currently available evidences of the toxicity management are mainly obtained from previous trials of CD19 targeted CAR T cells in B cell ALL or NHL, and are also applicable for patients with MM. Overall, the management of CAR T cell–related toxicity is dependent on its severity according to the ASTCT grading system (22, 95).

### General Management Strategies

Prior to CAR T cell therapy, the patients should be thoroughly screened as per clinical study protocols and/or local guidelines (93). The baseline characteristics may also have impact on the safety profile. CAR T cell therapy should only be given in patients with Eastern Cooperative Oncology Group (ECOG) performance score (PS) ≤ 2, close to normal end organ function, and acceptable blood count, and without any active bacterial, fungal, or viral infections (22, 85). In addition, high disease burden at baseline correlates with increased risk for CAR T cell–related toxicities such as CRS and ICANS (107). Therefore, a bridging therapy prior to CAR T cell infusion should be considered in these patients with the aim to “debulk” the tumor burden and to diminish the potential toxicities (115). Moreover, high CAR T cell dose can also increase the risk of toxicities (97). Split dose may be a strategy to circumvent this issue, especially in patients with high-risk features like EMD and PCL (115). Furthermore, preexisting neurological comorbidities may be risk factors for ICANS, and these patients need close neurological monitoring after CAR T cell infusion (26). To date, CNS involvement with MM is always an exclusion criterion in CAR T cell trials such that the safety data in this patient group are still missing (85).

The EMBT recommends a hospitalization of at least 14 days for the CAR T cell therapy. This facilitates a close monitoring of the patients after the treatment and, in case of necessity, a rapid medical intervention. However, shorter hospitalization or even outpatient management could also be considered, if specialist inpatient care was available for the patients within 30 min (22). ICU admission should be evaluated when the patients develop signs of ≥grade 2 CRS or any grade ICANS. The treating hematologist should also alert the referral neurologist, if the patients present neurological symptoms (87). Cytopenia following CAR T cell therapy can be managed using hematopoietic growth factors and transfusion of erythrocytes or thrombocytes. After the CAR T cell therapy, the patients should receive prophylaxis for *Pneumocystis jirovecii* and herpes virus according to the institutional practice for at least 6 and 12 months, respectively (116). In addition, antifungal and antibacterial prophylaxes can be considered in patients with prolonged cytopenia. At present, the role of antiviral prophylaxis for hepatitis B virus (HBV) or hepatitis C virus (HCV) in CAR T cell therapy remains unknown since these patients have been excluded from the currently available CAR T cell trials (22). However, if CAR T cell therapy would be integrated into the standard of care, this issue should be taken into account, as LDC and CAR T cells may potentially lead to HBV and/or HCV reactivation similar to that in patients treated with B cell depleting agent rituximab (117–119).

### Management of Cytokine Release Syndrome

Overall, supportive care is one of the major components in the management of CRS, as many cases of CRS are self-limiting and do not require any specific pharmacologic interventions (18). The patients should primarily be treated with antipyretics, oxygen, and intravenous fluids. Circulatory and/or respiratory support is indicated, when the patient shows hypotension and/or hypoxia, respectively (120). Moreover, the clinical and laboratory findings in CRS can overlap with that in sepsis caused by severe infections (121). Therefore, diagnostic tests such as laboratory examinations, imaging, blood and urine cultures etc. should be performed to identify or exclude an infection. Since CRS can mimic the clinical picture of neutropenic fever, a life-threatening emergency, prompt initiation of empiric broad-spectrum antibiotics is strongly recommended (22).

While supportive care is often sufficient for low grade CRS, patients with persistent or severe CRS do require specific pharmacologic interventions. The general concept of the specific CRS therapy is to neutralize the major cytokines and their receptors, or to reduce the cytokine production from CAR T cells or other immune cells. In August 2017, the IL-6 receptor (IL-6R) antagonist tocilizumab has been approved by the FDA for treatment of CRS. In a retrospective analysis of pooled data from nine clinical trials of anti-CD19 CAR T cell therapies in ALL or NHL, 69% of the patients showed CRS resolution within 14 days after the first dose of tocilizumab (122). At present, tocilizumab represents the first-line therapy for CAR T cell induced CRS. According to the current EBMT recommendations, tocilizumab should be given if fever ≥38°C persists three days, or if the patient exhibits hypoxia and/or hypotension. Usually, tocilizumab is administered intravenously at a dose of 8 mg/kg (maximum dose 800 mg), and can be repeated, if no improvement could be achieved after 8 h (22). Of note, Alvi et al. have reported that early administration of tocilizumab can reduce the risk of cardiovascular events following CAR T cell therapy (123). Importantly, tocilizumab does not increase the risk of clinically significant infections or infection density within 100 days (124). In patients who do not respond to tocilizumab, the second-line therapy with siltuximab, an IL-6 directed mAb, could be considered. However, there is only limited evidence for siltuximab therapy in (tocilizumab-refractory) CRS (41, 99, 125). Presently, a head-to-head comparison of tocilizumab and siltuximab is still missing. Another backbone of the CRS management is corticosteroid, which strongly diminishes the production and action of most cytokines (126). In the current EBMT guidelines, corticosteroids are recommended for patients with higher grade CRS, and are contraindicated in the absence of life-threatening events due to the potential CAR T cell suppression by them (22). By contrast, recent data from clinical trials of anti-CD19 or anti-CD22 CAR T cells in ALL have demonstrated that corticosteroids may mitigate the CAR T cell–related toxicities without influence on the efficacy (127). In addition, patients treated with corticosteroids have even shown significantly higher CAR T cell count in peripheral blood compared to the non-steroid group, suggesting that corticosteroids do not impair the CAR T cell expansion *in vivo* (128). In fact, the indication criteria for corticosteroid use in CRS vary widely among different centers. There are some other agents have been successfully applied in CRS patients, e.g., anti-IL-1 mAb anakinra (57) and TNF-α blocker etanercept (40). Tyrosine kinase inhibitors ruxolitinib and ibrutinib might also prevent CRS after CAR T cell therapy as suggested in preclinical studies (129, 130). However, robust data on their efficacy and safety in CRS is presently still pending. These treatment options could be considered as experimental salvage therapy for refractory CRS. We summarize the currently recommended management strategy for CRS in **Table 2** (22).

### Management of Immune Effector Cell-Associated Neurotoxicity Syndrome

As previously mentioned, there is a clear association between ICANS and CRS (98). The cytokine storm in CSF might be one of the major contributing factors to ICANS following CAR T cell therapy (97). Thus, the management strategy for ICANS is similar to that of CRS and, additionally, some specific neurologic issues should be noted.

ICANS is primarily managed with close monitoring and supportive care (87). The ICE score is a valuable tool to assess and monitor the patient’s neurologic status, which should be evaluated at least four times a day after the CAR T cell infusion (95). If the patients show any neurologic deficits regardless of grades, the treating physician should timely alert the referral neurologist and the local ICU. The head of the patient’s bed should be elevated to at least 30° to ensure a sufficient cerebral venous flow. Oral medication or nutrition should be switched to intravenous administration to avert aspiration (22).

It should be emphasized that the most of the patients are highly immunosuppressed and present thrombocytopenia after the CAR T cell therapy, indicating a markedly increased risk of atypical CNS infection and bleeding events (104). These are important differential diagnoses for ICANS. Indeed, progressive multifocal leukoencephalopathy (PML) caused by JC virus and fatal cerebral hemorrhage have already been observed in patients treated with CAR T cells (70, 131). Therefore, to identify or exclude other causes in patients with suspected ICANS, the patients should receive a neuroimaging, ideally MRI, and a diagnostic lumbar puncture for opening pressure and infection tests (22). In MRI of patients with ICANS, there are some characteristic patterns such as reversible T2 hyperintensities and focal cerebral edema in the bilateral thalami, external capsule, pons, and medulla (132). However, findings in neuroimaging are often nonspecific (133), or even normal in some cases (134). In addition, due to increased intracranial pressure and/or thrombocytopenia in some of the patients, lumbar puncture cannot be performed (104). Nonetheless, the treating physician should be aware of the aforementioned differential diagnoses as well as EMD progression of CNS or drug toxicity, which can likewise be life threatening. In addition to the above-discussed measures, if available, fundoscopy is recommended to exclude a papilledema. In patients with papilledema, acetazolamide could be considered (22). As papilledema is often a sign of increased ICP, hyperosmolar therapy with mannitol and/or hyperventilation are advised in these patients (22, 135).

Another major issue in ICANS is the management of seizure. After CAR T cell therapy, EEG is essential for the monitoring of patients with suspected ICANS, regardless of severity and presence of clinical seizure (22). The most common findings in EEG include diffuse or focal slowing, intermittent interictal epileptiform discharges and, in some cases, non-convulsive status epilepticus pattern (136). Especially, the patients with risk factors for ICANS, e.g., high tumor burden, EMD and PCL should receive seizure prophylaxis such as levetiracetam 750-mg bid (104). When the patients exhibit clinical seizure or non-convulsive status epilepticus in EEG, benzodiazepines or other anticonvulsive drugs such as valproate, phenytoin, barbiturate, lacosamide, and propofol can be given to terminate seizures (68, 134, 136).

As of September 2020, there is still no approved agent for specific ICANS therapy. ICANS patients with concurrent CRS are primarily managed with tocilizumab (22). However, tocilizumab is a large mAb that cannot penetrate the BBB in relevant concentration. Evidence from primate model has suggested that intrathecal administration of tocilizumab might be an option to overcome the BBB (137). More importantly, tocilizumab binds to IL-6R and, therefore, may even cause an increased level of IL-6 after tocilizumab use (96). This phenomenon may potentially aggravate ICANS. Indeed, Frigault et al. have recently reported that patients receiving tocilizumab were more likely to develop ICANS in comparison with those without tocilizumab (124). Currently, the role of tocilizumab in ICANS management is still controversial, and further investigations are required at this point. In contrast to tocilizumab, siltuximab antagonizes IL-6 and has a smaller molecular size such that it can pass the BBB (138–140). However, studies directly comparing tocilizumab with siltuximab in ICANS are still not available. Currently, corticosteroids represent the mainstays for ICANS managements due to their immunosuppressive effects. Corticosteroids are typically indicated in ICANS ≥grade 2 (22). In anti-BCMA CAR T cell trials in MM, the patients have shown a rapid resolution of ICANS after high dose corticosteroid therapy (39, 56, 60). On the other hand, the optimal duration of steroid therapy is still undefined. In a recent study of Karschnia et al., a shorter course of steroid treatment (<7 days) does not significantly alter the survival outcome of ICANS patients when compared with longer steroid use (≥7 days). Interestingly, prolonged steroid therapy of ≥10 days indicates a significantly inferior overall survival (OS) in comparison with <10 days steroid, probably due to the severity of ICANS itself (134). Experience with steroid refractory ICANS is lacking, and these patients have often an unfavorable prognosis. The IL-1 antibody anakinra might be a salvage option, as it can cross the BBB and can prevent ICANS in mouse model (141, 142). Anecdotal reports have suggested that additional triple intrathecal chemotherapy with cytarabine, methotrexate, and hydrocortisone could be effective in steroid refractory ICANS (143). Moreover, “re-lymphodepleting” with high dose cyclophosphamide (e.g., 1.5 g/m^2^) in addition to steroid may also have some efficacy (41). In summary, the pharmacologic therapy for ICANS is still a matter of debate. The current recommendations for ICANS management are shown in **Table 3**. Importantly, the ICANS monitoring and management strategies are mainly based on protocols of several clinical trials that have been performed at our center. Today, there is still no international standard of ICANS management, and the institutional practice might be not necessarily applied universally.

## Conclusions and Future Considerations

In recent years, CAR T cell has opened up a new era of the immunotherapy for MM. This valuable approach has the potential to further improve the survival outcome of MM patients. On the other hand, despite the impressive efficacy and marked progress in the development, this young research field is still in its “puberty”, with a variety of unsettled issues, e.g., financial burden and the management of toxicities (86). A recent analysis from the USA has yielded an estimated total cost of $454 611 for one course of CAR T cell therapy, which may restrict the availability of this novel therapy, in particular for countries with limited resources (144). Although the data from clinical trials have demonstrated only few toxic deaths related to CAR T cell in MM patients, incidences of therapy-related AEs such as CRS and ICANS are quite high, with fever ≥38°C being even the “standard” event after CAR T cell infusion. Fortunately, the cytokine storm with fever also indicates the remarkable efficacy of CAR T cells and, thus, it could be accepted that the MM would disappear after suffering from several days of high fever. However, CRS and/or ICANS may progress to a life-threatening event and require ICU admission, which may further prolong the hospitalization and increase the cost of the treatment. Taken together, the cost of CAR T cell therapy may be reduced by optimizing the toxicity management. To overcome these limitations, the following aspects have been considered: 1) modifications of the CAR T cells, and 2) pharmacologic intervention to attenuate CRS and ICANS.

T cells can be engineered to express both CAR and an additional antigen such as CD20 or truncated epidermal growth factor receptor (EGFRt). In this way, these CAR T cells can be antagonized using already approved mAbs rituximab or cetuximab, if the treatment causes unacceptable toxicity (145, 146). For patients with MM, clinical trial investigating anti-BCMA CAR T cells with EGFRt co-expression (EGFRt/BCMA-41BBz) is currently ongoing (NCT03070327). Similarly, a suicide gene, e.g., inducible safety switch caspase 9 (iCasp9) can be incorporated into the CAR T cell. In patients experiencing severe toxicities after CAR T cell therapy, iCasp9 can be activated by the small molecule dimerizer drug AP1903, initiating a signaling cascade leading to rapid apoptosis of the CAR T cells (147). Another strategy is to design an “all-purpose” fluorescein isothiocyanate (FITC) targeted CAR T cell that can be activated only if a bispecific adapter links it to tumor cells (148). Preclinical data in mouse model have demonstrated that CRS-like toxicity can be regulated by controlling the dose of the bispecific adapter, which connects the tumor cell and anti-FITC CAR T cell (149). However, rigorous evidence in human is currently not available.

With tocilizumab and/or corticosteroid remaining the backbone of the management of CAR T cell toxicity, some alternative agents have been evaluated in preclinical setting. For instance, GM-CSF inhibition using lenzilumab can attenuate CRS and ICANS without impairment of the CAR T cell function (150). Moreover, as endothelial damage is regarded as a major “driver” in CRS and/or ICANS, endothelial protection using defibrotide, an approved agent for treatment of veno-occlusive disease following SCT, might be an option for CAR T cell–related toxicities (151, 152). Clinical investigation is currently underway (89). Recently, Mestermann et al. have reported that the tyrosine kinase inhibitor dasatinib can reversibly inhibit the cytolytic activity, cytokine production, and proliferation of CAR T cells *in vitro* and *in vivo*, suggesting that dasatinib could potentially be an option to alleviate CRS and/or ICANS after CAR T cell therapy (153).

In summary, CAR T cell is now revolutionizing the treatment of MM with amazing efficacy and acceptable safety profile. Elucidating the underlying pathophysiology may provide novel rationales for pharmacologic intervention of CAR T cell–related toxicities. Improvement of the safety of CAR T cells can enable widespread use of this promising therapy approach, and can bring hopes for more patients with MM. Further studies in this research field are highly warranted.

## Author Contributions

XZ wrote the initial manuscript. LR, KK, SD, MH, and HE edited and approved the final manuscript. All authors contributed to the article and approved the submitted version.

## Funding

This publication was supported by the Open Access Publication Fund of the University of Wuerzburg. LR is funded by the Deutsche Krebshilfe via the MSNZ programme.

## Conflict of Interest

MH is listed as an inventor on patent applications and granted patents related to CAR-T technologies that have been filed by the Fred Hutchinson Cancer Research Center, Seattle, WA and by the University of Würzburg, Würzburg, Germany.

MH received honoraria from Celgene/BMS, Janssen, Kite/Gilead.

The remaining authors declare that the research was conducted in the absence of any commercial or financial relationships that could be construed as a potential conflict of interest.
